# Successful Re-challenge of zongertinib following erythema multiforme-like drug eruption in a patient with HER2-mutant non-small cell lung cancer

**DOI:** 10.1016/j.rmcr.2026.102444

**Published:** 2026-06-02

**Authors:** Kodai Kawamura, Kaori Kubota, Kazuya Ichikado

**Affiliations:** Division of Respiratory Medicine, Saiseikai Kumamoto Hospital, Kumamoto, Japan

**Keywords:** Zongertinib, HER2-Mutant non-small cell lung cancer, Drug eruption, Erythema multiforme, Re-challenge, Tyrosine kinase inhibitor

## Abstract

**Background:**

Zongertinib is a novel, oral, HER2-selective tyrosine kinase inhibitor (TKI) that spares wild-type EGFR. While it has a favorable safety profile, severe cutaneous adverse events (scAEs) can occur, especially in the post-immunotherapy setting.

**Case presentation:**

A 55-year-old female with HER2-mutant non-small cell lung cancer (NSCLC) was treated with zongertinib as second-line therapy following chemo-immunotherapy with pembrolizumab. By day 14 of zongertinib treatment, she developed a high fever (39.4 °C), generalized targetoid eruptions, and mucosal involvement, leading to a diagnosis of erythema multiforme (EM)-like drug eruption. Although zongertinib was initially discontinued and a reduced-dose re-challenge failed, a subsequent step-up protocol—starting with every-other-day (QOD) followed by daily (QD) dosing—was successful. Serial chest radiographs confirmed significant tumor shrinkage in the left lung, achieving a partial response without recurrence of severe scAEs.

**Conclusion:**

A cautious dose-escalation strategy can allow for the continuation of zongertinib even after severe EM-like eruptions, preserving a potent treatment option.

## Abbreviations

EM: Erythema multiforme
HER2: Human epidermal growth factor receptor 2
ICI: Immune checkpoint inhibitor
NSCLC: Non-small cell lung cancer
PR: Partial response
QD: Quaque die (once daily)
QOD: Quaque altera die (every other day)
scAE: Severe cutaneous adverse event
TKI: Tyrosine kinase inhibitor

## 1 Introduction

Zongertinib is a highly selective, irreversible oral TKI specifically designed to target HER2 tyrosine kinase domain mutations while sparing wild-type EGFR [1]. This selectivity significantly reduces EGFR-mediated toxicities, such as acneiform rash, which are common with earlier-generation pan-HER inhibitors [2]. However, immune-mediated hypersensitivity reactions like erythema multiforme (EM) remain a clinical concern [3], particularly when administered sequentially after immune checkpoint inhibitors (ICIs). We report a case of successful zongertinib re-challenge using a step-up protocol following a severe EM-like eruption, highlighting the impact of prior pembrolizumab therapy on the severity of the reaction.

## 2 Case Presentation

A 55-year-old female was diagnosed with HER2-mutant NSCLC. First-line therapy consisted of carboplatin, pemetrexed, and pembrolizumab, achieving a partial response (PR). Following disease progression, zongertinib (120 mg daily) was initiated as second-line therapy.

**Fig. 1:**
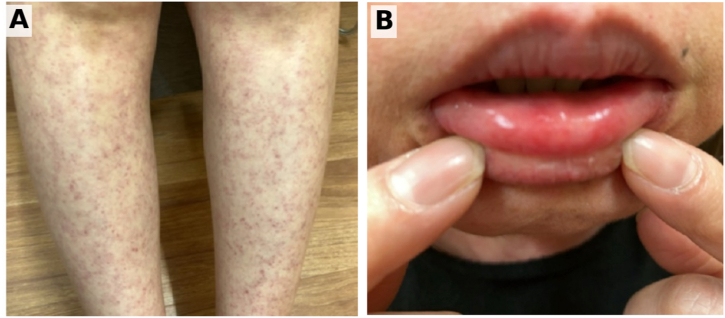
Clinical manifestations of zongertinib-induced erythema multiforme (A) Symmetric targetoid erythematous macules on the lower extremities. (B) Hemorrhagic crusting and erosions on the lips.

On day 12 of zongertinib treatment, the patient developed a generalized rash. By day 14, she presented with a high fever (39.4 °C) and symmetric targetoid erythematous lesions on the neck, forearms, thighs, and ankles. The eruption rapidly progressed to involve the entire body, with emergent mucosal involvement including hemorrhagic crusting of the lips and oral erosions (Fig. 1). Laboratory findings at the peak of the eruption showed elevated C-reactive protein (1.47 mg/dL) and transient cytopenia.

Zongertinib was immediately discontinued, and systemic prednisolone (30 mg/day) was initiated. The fever and cutaneous lesions improved significantly within days. Prednisolone was then tapered to 20 mg/day after four days and subsequently discontinued over the following two weeks as the rash resolved.

**Fig. 2:**
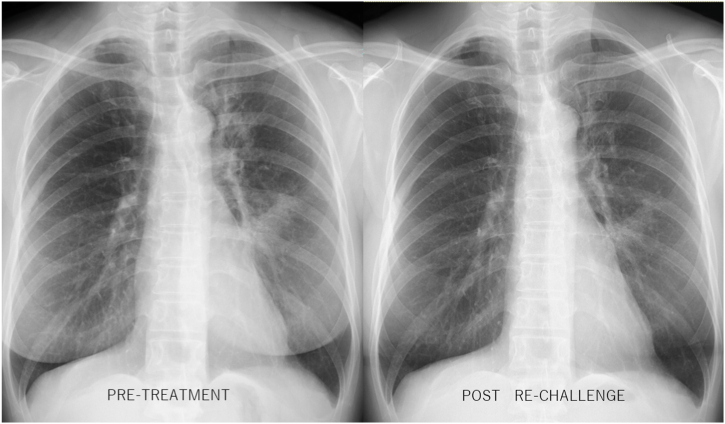
Radiographic response to zongertinib. Chest radiographs showing significant reduction of the lung tumor following the successful re-challenge of zongertinib.

Two weeks after the complete resolution of the skin lesions and discontinuation of prednisolone, a re-challenge with zongertinib at the reduced dose (60 mg daily) was attempted. However, this resulted in an immediate recurrence of generalized erythema within 24 hours, necessitating another treatment interruption. One week later, a more cautious step-up protocol was initiated. Zongertinib (60 mg) was administered every other day (QOD). After confirming no recurrence for eight days, the regimen was increased to 60 mg daily (QD). This step-up approach was well-tolerated with only transient, mild erythema. Serial chest radiographs confirmed significant tumor shrinkage in the left lung field, consistent with a partial response (PR) (Fig. 2). The patient continues to receive zongertinib daily without recurrence of severe scAEs.

## 3 Discussion

A distinctive feature of this case is the development of a severe EM-like eruption despite the use of zongertinib. Unlike previous-generation inhibitors, zongertinib is EGFR-sparing, which typically results in fewer cutaneous toxicities. The occurrence of Grade 3 EM-like eruption suggests that the pathophysiology was not a direct off-target effect on EGFR, but a potent immune-mediated hypersensitivity reaction.

We hypothesize that the prior administration of pembrolizumab played a decisive role. Sequential therapy with ICIs followed by TKIs markedly increases the risk of severe immune-related adverse events [4,5]. The prolonged half-life of pembrolizumab likely “primed” the immune system, leading to an exaggerated T-cell response upon zongertinib initiation.

Furthermore, while zongertinib spares wild-type EGFR, its potent inhibition of HER2 in the skin may contribute to inflammatory cascades, as HER2 is vital for epidermal stem cell homeostasis [6]. The synergy between ICI-induced immune priming and HER2 inhibition likely created a “double-hit” mechanism.

Given zongertinib's efficacy, avoiding permanent discontinuation is crucial. Our success with a QOD-to-QD step-up protocol demonstrates that clinical desensitization is achievable even after severe hypersensitivity to selective TKIs [[7], [8], [9], [10], [11]]. To the best of our knowledge, this is the first reported case of successful desensitization to zongertinib. Table 1 summarizes published case reports of successful desensitization following hypersensitivity reactions to kinase inhibitors and HER2-targeted therapies. This approach may induce peripheral immune tolerance, allowing for the safe re-introduction of a critical therapeutic agent. The subsequent radiographic improvement observed on follow-up chest radiographs underscores the clinical benefit of maintaining zongertinib through this individualized strategy.

**Table 1:** Published case reports of successful desensitization following hypersensitivity reactions to kinase inhibitors and HER2-targeted therapies.

Author (Year)	Drug	Drug Class	Indication	Reaction Type	Desensitization Protocol	Outcome
Anderson et al. (2020)	Alectinib	ALK TKI	ALK + NSCLC	Severe drug rash (Grade 3)	Gradual dose escalation from sub-therapeutic starting dose; modified published protocol	Successful; no recurrence of skin toxicity; treatment continued
Ben-Ami et al. (2018)	Imatinib	BCR-ABL/PDGFR TKI	Metastatic dermatofibrosarcoma protuberans	DRESS syndrome	Step-wise escalation: 50 mg to 400 mg/day under allergy clinic supervision; concurrent prednisone taper	Successful; no DRESS recurrence; 6 additional months of clinical benefit
Bumbacea et al. (2022)	Dabrafenib	BRAF inhibitor	BRAF V600E + metastatic melanoma	Hypersensitivity reaction (skin, systemic)	Two protocols: (1) Rapid 3-day escalation; (2) Slow 14-day escalation. Both reached full daily dose.	Both tolerated; no recurrent hypersensitivity; combined therapy continued
Robinson et al. (2023)	Trastuzumab	Anti-HER2 monoclonal antibody	HER2+ locally advanced breast cancer	Severe anaphylaxis	Multidisciplinary oncology-allergy clinic protocol; standardized stepwise infusion rate escalation	Successful; trastuzumab continued as first-line therapy
Present case	Zongertinib	HER2 TKI (exon 20-selective)	HER2-mutant NSCLC	Erythema multiforme-like drug eruption (Grade 3)	Step-up rechallenge: QOD dosing (60 mg) for 8 days, then QD (60 mg/day)	Successful; no recurrence of eruption; partial response maintained

ALK, anaplastic lymphoma kinase; BRAF, B-Raf proto-oncogene; DRESS, drug reaction with eosinophilia and systemic symptoms; HER2, human epidermal growth factor receptor 2; NSCLC, non-small cell lung cancer; PDGFR, platelet-derived growth factor receptor; QD, once daily; QOD, every other day; TKI, tyrosine kinase inhibitor.

## Ethics statement

Informed consent was obtained from the patient for the publication of this case report and accompanying images. Ethical approval was waived by our institutional review board as this is a case report of a single patient.

## Data availability statement

Data sharing is not applicable to this article as no new datasets were created or analyzed. All relevant clinical data are included within the published article.

## Declaration of generative AI and AI-assisted technologies in the manuscript preparation process

During the preparation of this work, the authors used Gemini (Google) in order to improve the English language and to structure the manuscript. After using this tool/service, the authors reviewed and edited the content as needed and take full responsibility for the content of the publication.

## Funding information

None.

## CRediT authorship contribution statement

**Kodai Kawamura:** Conceptualization, Data curation, Investigation, Visualization, Writing – original draft, Writing – review & editing. **Kaori Kubota:** Investigation, Writing – review & editing. **Kazuya Ichikado:** Investigation, Supervision, Writing – review & editing.

## Declaration of competing interest

The authors declare that they have no known competing financial interests or personal relationships that could have appeared to influence the work reported in this paper.

## References

[1] Heymach J.V., Opdam F., Barve M., Tu H.-Y., Wu Y.-L., Berz D., Schröter L., Botilde Y., Sadrolhefazi B., Serra J., Yoh K., Yamamoto N. (2025). HER2-selective tyrosine kinase inhibitor, zongertinib (BI 1810631), in patients with advanced/metastatic solid tumors with HER2 alterations: a phase Ia dose-escalation study. J. Clin. Oncol..

[2] Lacouture M.E., Sibaud V., Gerber P.A. (2021). Prevention and management of dermatological toxicities related to HER2-targeted therapies. Lancet Oncol..

[3] Ying S., Gu X., Lian B. (2022). Erythema multiforme induced by pyrotinib in a patient with HER2-positive metastatic breast cancer: a case report. J. Chemother..

[4] Schoenfeld A.J., Arbour K.C., Mezquita L. (2019). Severe immune-related adverse events are common with sequential PD-(L)1 blockade and osimertinib. Ann. Oncol..

[5] Oshima Y., Tanimoto T., Yuji K., Tojo A. (2018). EGFR-TKI-associated interstitial pneumonitis in nivolumab-treated patients with non-small cell lung cancer. JAMA Oncol..

[6] Nanba D., Toki F., Matsushita N. (2013). EGFR-system-mediated self-renewal of mammary epithelial stem cells. J. Cell Biol..

[7] Bareschino M.A., Schettino C., Rossi A. (2010). Successful desensitization to gefitinib in a patient with EGFR-mutated non-small-cell lung cancer. J. Thorac. Oncol..

[8] Anderson B.E., Luczak T.S., Ries L.M., Hoefs G.E., Silva-Benedict A.C. (2020). Successful alectinib desensitization in a patient with anaplastic lymphoma kinase-positive adenocarcinoma of the lung and alectinib-induced drug rash. J. Oncol. Pharm. Pract..

[9] Ben-Ami E., Castells M.C., Connell N.T., Rutherford A.E., Thornton K.A. (2018). Imatinib-induced drug reaction with eosinophilia and systemic symptoms in solid tumors: a patient with dermatofibrosarcoma protuberans and successful desensitization management. Anti Cancer Drugs.

[10] Bumbacea R.S., Ali S., Corcea S.L., Jinga D.C., Spiru L. (2022). Successful dabrafenib desensitization protocols in a patient with metastatic melanoma. Medicina (Kaunas).

[11] Robinson M., Geirnaert M., Anderson B., McKibbin L. (2023). Canada's first joint oncology-allergy clinic: successful desensitization to trastuzumab following severe anaphylactic reaction. Curr. Oncol..

